# Response to García-Nieto et al. Comments on Beamer et al. Association of Children’s Urinary CC16 Levels with Arsenic Concentrations in Multiple Environmental Media. *Int. J. Environ. Res. Public Health* 2016, *13*, 521

**DOI:** 10.3390/ijerph13100978

**Published:** 2016-10-04

**Authors:** Paloma I. Beamer, Walter T. Klimecki, Miranda Loh, Yoshira Ornelas Van Horne, Anastasia J. Sugeng, Nathan Lothrop, Dean Billheimer, Stefano Guerra, Robert Clark Lantz, Robert A. Canales, Fernando D. Martinez

**Affiliations:** 1 Asthma and Airway Disease Research Center, 1501 N. Campbell Ave., University of Arizona, Tucson, AZ 85724, USA; dean.billheimer@arizona.edu (D.B.); stefano@email.arizona.edu (S.G.); fernando@arc.arizona.edu (F.D.M.); 2 Mel and Enid Zuckerman College of Public Health, University of Arizona, 1295 N. Martin Ave., Tucson, AZ 85724, USA; klimecki@pharmacy.arizona.edu (W.T.K.); miranda.loh@gmail.com (M.L.); yornelas@email.arizona.edu (Y.O.V.H.); asugeng@email.arizona.edu (A.J.S.); lothrop@email.arizona.edu (N.L.); lantz@email.arizona.edu (R.C.L.); rcanales@email.arizona.edu (R.A.C.); 3 Department of Chemical and Environmental Engineering, University of Arizona, 1133 E. James E. Rogers Way, Tucson, AZ 85721, USA; 4 Department of Pharmacology and Toxicology, College of Pharmacy, University of Arizona, P.O. Box 210207, Tucson, AZ 85724, USA; 5 Institute of Occupational Medicine, Research Avenue North, Riccarton, Edinburgh EH14 4AP, UK; 6 Department of Cellular and Molecular Medicine, University of Arizona, P.O. Box 245044, Tucson, AZ 85724, USA

We would like to thank the editors for providing us with the opportunity to respond to the points raised by Dr. García Nieto.

Our group and others have demonstrated that lower levels of circulating CC16 are associated with lower lung function, greater airflow limitation, and increased mortality [1,2]. More recently, work from our group has demonstrated that lower levels of circulating CC16 at six years of age is associated with decreased lung function by the teenage years in multiple birth cohorts [3]. Another group has reported that the odds of a child having asthma decreased with increasing CC16 levels in their urine, and their forced vital capacity increased with increasing levels of urinary CC16 [4]. Similarly, in a prospective cohort, higher urinary CC16 levels in infants at the time of an acute lower respiratory tract infection were associated with decreased odds of subsequent childhood wheeze [5]. Taken together, these findings indicate that CC16 may be an important biomarker of early life epithelial and airway damage from potentially preventable environmental exposures.

Because others have demonstrated that decreased levels of CC16 are associated with increasing arsenic exposure [6,7,8], we sought to determine if this association persisted in children. We reported that decreased levels of urinary CC16 in children were most strongly associated with the concentration of arsenic in their soil and that this may demonstrate that localized arsenic exposure in the lungs could damage the airway epithelium [9].

Arsenic exposure is associated with adverse health effects in virtually every physiological system in the body [10], including the respiratory system and, as Dr. García Nieto indicates, the renal system. Specifically, he highlights that arsenic exposure can cause proximal tubular dysfunction and thus reduce the amount of CC16 that can be reabsorbed by the kidneys, resulting in increased urinary CC16 levels in those with increased arsenic exposure [11]. However, individuals exposed to arsenic have reduced levels of CC16 in their serum, and it is not clear if arsenic-induced proximal tubular dysfunction would compensate for that difference.

In occupational cohorts, arsenic exposure was associated with decreased serum CC16 and increased urinary β2-microglobulin, yet no significant differences in serum β2-microglobulin were reported [6,7]. Unfortunately, we did not have serum samples to measure CC16 for this population. However, none of the children in our study reported any history of kidney injury, disease, or failure. If their arsenic exposure had reduced their kidneys’ ability to reabsorb CC16 from their urine, then, if anything, we have underestimated the potential effect of arsenic exposure on CC16 production in the body.

We should, and do, acknowledge alternative interpretations of the data. This is particularly important because, even though there have been multitudinous studies of arsenic-exposed human populations, there have been relatively few studies involving urinary CC16 in the context of arsenic exposure. Future studies that measure both CC16 in serum and urine, as well as arsenic exposure, will be needed to better understand our findings and elucidate the competing effects of arsenic toxicity of the lungs and the kidneys on CC16 levels in children’s urine. Our hope is that our results encourage further investigation of the relationship between arsenic exposure and CC16 in larger populations, particularly between high- and low-exposure areas.

Based upon his own work, Dr. García Nieto’s second point is that detectable levels of CC16 in urine are very low among healthy children [11,12,13]. We also had a large number of undetectable values of CC16 in children’s urine with the assay we used (Human Uteroglobulin Quantikine ELISA kit, R & D Systems, Minneapolis, MN, USA) (Limit of Detection [LOD] = 0.8 ng/mL). We do not dispute that healthy children may have low urinary CC16 levels. However, this does not preclude the finding that, in our population, particularly in light of the number of censored values, there was a negative association between the concentration of arsenic in the children’s soil and the levels of urinary CC16.

Unfortunately, one of Dr. García-Nieto’s studies is not yet available from the journal [13,14], and the other study does not report the LOD of the ELISA kit used, or how censored values were treated in the analysis. In addition, children with respiratory disease, who may have even lower levels of urinary CC16 than otherwise healthy children, were excluded from that study [12]. Thus, it is difficult to fully compare results. However, it is noteworthy that, despite the low CC16 concentrations in urine, previous studies that measured urinary CC16 levels using different ELISA kits were also able to demonstrate inverse associations of urinary CC16 levels with concurrent asthma and lung function deficits in school-age children [4] and with subsequent risk of childhood wheezing in infants with lower respiratory tract infections [5].

In summary, both serum and urinary CC16 demonstrate promise as biomarkers that could be developed and validated as measures of early effects of lung epithelium injury or pulmonary permeability. However, given the complexities of this interesting protein, as discussed in Dr. García-Nieto’s points, multiple studies are needed to better understand the role of environmental exposures, including arsenic, and disease in relation to its levels in serum and urine. We look forward to our study contributing to this process.
